# Characterization of Oxidative Stress in Various Tissues of Diabetic and Galactose-fed Rats

**DOI:** 10.1155/EDR.2001.211

**Published:** 2001

**Authors:** Robert M. Strother, Tonya G. Thomas, Mary Otsyula, Ruth A. Sanders, John B. Watkins III

**Affiliations:** 1 Medicat Sciences Indiana University School of Medicine Jordan Hall 104 Bloomington IN 47405-7005 USA; 2 Faculty of Health Sciences Moi University Eldoret Kenya

## Abstract

Rats fed a galactose-rich diet have been used for several years as a model for diabetes to study, particularly in the eye, the effects of excess blood hexoses. This study sought to determine the utility of galactosemia as a model for oxidative stress in extraocular tissues by examining biomarkers of oxidative stress in galactose-fed rats and experimentally-induced diabetic rats. Sprague-Dawley rats were divided into four groups: experimental control; streptozotocin-induced diabetic; insulin-treated diabetic; and galactose-fed. The rats were maintained on these regimens for 30 days, at which point the activities of catalase, glutathione peroxidase, glutathione reductase, and superoxide dismutase, as well as levels of lipid peroxidation and reduced and oxidized glutathione were determined in heart, liver, and kidney. This study indicates that while there are some similarities between galactosemic and diabetic rats in these measured indices of oxidative stress (hepatic catalase activity levels and hepatic and renal levels of oxidized glutathione in both diabetic and galactosemic rats were significantly decreased when compared to normal), overall the galactosemic rat model is not closely parallel to the diabetic rat model in extra-ocular tissues. In addition, several effects of diabetes (increased hepatic glutathione peroxidase activity, increased superoxide dismutase activity in kidney and heart, decreased renal and increased cardiac catalase activity) were not mimicked in galactosemic rats, and glutathione concentration in both liver and heart was affected in opposite ways in diabetic rats and galactose- fed rats. Insulin treatment reversed/prevented the activity changes in renal and cardiac superoxide dismutase, renal and cardiac catalase, and hepatic glutathione peroxidase as well as the hepatic changes in lipid peroxidation and reduced and oxidized glutathione, and the increase in cardiac glutathione. Thus, prudence should be exercised in the use of experimentally galactosemic rats as a model for diabetes until the correspondence of the models has been more fully characterized.


*Int. Jnl. Experimental Diab. Res.*, Vol. 2, pp. 211–216
Reprints available directly from the publisher
Photocopying permitted by license only

© 2001 OPA (Overseas Publishers Association) N.V.
Published by license under
the Harwood Academic Publishers imprint,
part of Gordon and Breach Publishing,
a member of the Taylor & Francis Group.
Printed in the U.S.A.

### Characterization of Oxidative Stress in Various Tissues of Diabetic and Galactose-fed Rats

ROBERT M. STROTHER[a], TONYA G. THOMAS[a], MARY OTSYULA[b], RUTH A. SANDERS[a] and JOHN B. WATKINS III[a,*]

[a]*Medical Sciences, Indiana University School of Medicine, Jordan Hall 104, Bloomington, IN 47405-7005;*
[b]*Faculty of Health Sciences, Moi University, Eldoret, Kenya*

*(Received 27 March 2001; Revised 2 July 2001; In final form 3 August 2001)*

**Rats fed a galactose-rich diet have been used for several years as a model for diabetes to study, particularly in the eye, the effects of excess blood hexoses. This study sought to determine the utility of galactosemia as a model for oxidative stress in extra-ocular tissues by examining biomarkers of oxidative stress in galactose-fed rats and experimentally-induced diabetic rats. Sprague-Dawley rats were divided into four groups: experimental control; streptozotocin-induced diabetic; insulin-treated diabetic; and galactose-fed. The rats were maintained on these regimens for 30 days, at which point the activities of catalase, glutathione peroxidase, glutathione reductase, and superoxide dismutase, as well as levels of lipid peroxidation and reduced and oxidized glutathione were determined in heart, liver, and kidney. This study indicates that while there are some similarities between galactosemic and diabetic rats in these measured indices of oxidative stress (hepatic catalase activity levels and hepatic and renal levels of oxidized glutathione in both diabetic and galactosemic rats were significantly decreased when compared to normal), overall the galactosemic rat model is not closely parallel to the diabetic rat model in extra-ocular tissues. In addition, several effects of diabetes (increased hepatic glutathione peroxidase activity, increased superoxide dismutase activity in kidney and heart, decreased renal and increased cardiac catalase activity) were not mimicked in galactosemic rats, and glutathione concentration in both liver and heart was affected in opposite ways in diabetic rats and galactose-fed rats. Insulin treatment reversed/prevented the activity changes in renal and cardiac superoxide dismutase, renal and cardiac catalase, and hepatic glutathione peroxidase as well as the hepatic changes in lipid peroxidation and reduced and oxidized glutathione, and the increase in cardiac glutathione. Thus, prudence should be exercised in the use of experimentally galactosemic rats as a model for diabetes until the correspondence of the models has been more fully characterized.**

*Keywords*: Galactose; Glucose; Diabetes; Galactosemia; Rat; Streptozotocin

### INTRODUCTION

The defining property of diabetes mellitus (DM) is the inability of the body to regulate glucose metabolism. This inability arises as a result of either insufficient or nonexistent insulin production, as in Type I DM, or tissue insensitivity to insulin, as in Type II DM. This disruption of normal metabolic regulatory processes causes both short-term complications, such as hyperglycemia, and long-term complications, such as arteriosclerosis and microangiopathies.[1] While both behavioral modification and medication have had success in controlling aspects of the disease, many of the chronic complications

*Corresponding author. Tel.: 812-855-3201, Fax: 812-855-4436, e-mail: watkins@indiana.edu continue to elude treatment. Notably, DM patients are at significantly higher risk for cardiovascular disease, retinopathy, peripheral neuropathy, and nephropathy.[1]

Research has long implicated free radical oxidative stress resulting from hyperglycemia as one source of these long-term complications.[2–4] Circulating glucose, unable to enter tissues due to the deficit of or insensitivity to insulin, collects in excess in both the blood and non-insulin dependent tissues. Oxidative stress may be created as the overabundant hexoses engage in metal catalyzed oxidation,[5] participate in glycation reactions with proteins and lipoproteins,[6] or enter the polyol pathway resulting in the conversion of glucose into sorbitol and the release of free radicals.[7]

For nearly two decades, experimentally induced hypergalactosemia has been used as a model to study the morphological and biochemical changes that retinal tissue undergoes as a result of high blood sugars.[8,9] The retinopathy that develops under these conditions is morphologically similar to that which occurs in diabetes,[10,11] and the stability of the hypergalactosemic state facilitates long-term studies, which are difficult in experimentally diabetic animals. The elegance of this model lies in looking only at the effects of high blood sugar, excluding the complex array of metabolic and hormonal imbalances that arise in DM.[10] In spite of the great utility of this model in mimicking diabetic retinopathy, the whole-animal effects of hypergalactosemia have been less thoroughly examined. Although galactosemia has been used for years in biochemical and morphological studies in many tissues, investigations into the interactions of galactosemia and oxidative stress in extra-ocular tissues are minimal in the literature, and it is unclear whether the galactose-fed rat can serve as a model for the oxidative stresses in DM. This study hypothesizes that focusing solely on excess hexose, as in experimental galactosemia, does not provide a reasonable model of the oxidative stresses experienced in various tissues by diabetic rats.

### MATERIALS AND METHODS

### Materials and Animals

All chemicals were purchased from Sigma (St. Louis, MO). Male Sprague-Dawley rats (175–200 g) were procured from Harlan Sprague Dawley (Indianapolis, IN). After 7 days acclimatization, the rats were randomly divided into four treatment groups of 15 rats. One group was left untreated as normal controls. In two groups, diabetes was induced by a bolus intravenous STZ injection into a saphenous vein (100 mg/kg in freshly prepared 10 mM sodium citrate, pH 4.5). One group of diabetic rats was treated with insulin (2–4 units PZI insulin/day sc) from Eli Lilly (Indianapolis, IN). The fourth group of 15 was fed a diet of 50% galactose (Dyets, Bethleham, PA). All other rats were given Purina Rat Chow (No. 5012, St. Louis, MO) and water *ad libitum*. Diabetic rats having serum glucose levels $<350$ mg/dl, as determined by Sigma glucose (HK) kit, were excluded from the study. Animal experimentation and husbandry adhered to the U.S. Public Health Service guidelines.[12]

### Tissue Collection and Assay

Thirty days after the beginning of treatments, animals were anesthetized with halothane (2–4%, ih). Blood was drawn by cardiac puncture for terminal blood glucose concentrations and for quantitation of hemoglobin A1c (Hb1Ac) levels with Sigma Kit 441-B. Liver, kidney, and heart were excised and immediately frozen to $-70$°C. Frozen tissue from each rat was homogenized (5% w/v) in ice-cold 0.1 M Tris-HCl buffer (pH 7.4) and assayed for degree of lipid peroxidation by measuring the concentration of thiobarbituric acid reactive substances (TBARS).[13] The remaining homogenates were centrifuged at 100,000 × g for 1 hour, and supernatants from each liver, kidney, and heart were assayed for activity levels of catalase,[14] superoxide dismutase,[15] glutathione peroxidase,[16] and glutathione reductase.[17] Another 0.25 g aliquot of each tissue was homogenized in 3.75 ml ice-cold 0.1 M sodium phosphate-5 mM EDTA (pH 8.0), then 1 ml 25% phosphoric acid was added. After vortexing for 10 sec, the samples were centrifuged at 100,000 × g for 30 min. The resulting supernatants were assayed for concentrations of both reduced (GSH) and oxidized (GSSG) glutathione.[18] Protein concentration in all samples was determined by the method of Lowry.[19]

### Statistical Analysis

For each tissue, means and standard errors of the means were calculated for all groups. Data were analyzed by one-way analysis of variance followed by Duncan's test. Significance was set at $p < 0.05$. In the table and figures, all experimental groups (diabetic, insulin-treated diabetic, and galactose-fed) were compared to the untreated control group and significant differences are indicated by *

### RESULTS

Table I shows general characteristics of the experimental animals. Overall body weight of diabetic rats and galactosemic rats was decreased in comparison to that of normal rats. However, because the liver weight of the galactosemic rats was also significantly lower than normal, the liver-to-body-weight ratio, while elevated in diabetic rats, was normal in galactose-fed rats. As expected, the blood glucose concentrations in diabetic rats were significantly higher than in normal controls, and there was a parallel increase in glycosylated hemoglobin, as measured by HbA1c. The galactosemic rats showed no significant increase or decrease in blood glucose, but the apparent increase in HbA1c was not significant at $p < 0.05$. In retrospect this is plausible because, while glycosylation secondary to excess glucose is best measured with HbA1c, the glycosylation secondary to galactosemia is more detectable on the HbA1a and HbA1b fractions.[20] Insulin treatment reversed/prevented the diabetes-induced changes in body weight, liver/body weight ratio, serum glucose concentrations and HbA1c levels.

Comparison of biomarkers of oxidative stress within the rats revealed some trends. Hepatic catalase activity levels in both diabetic and galactosemic rats were significantly decreased when compared to normal (Fig. 1). Hepatic and renal levels of GSSG were significantly diminished compared to normal in both diabetic and galactosemic rats (Fig. 2).

However, several effects of diabetes (increased glutathione peroxidase activity in liver, increased superoxide dismutase activity in kidney and heart, decreased renal and increased cardiac catalase activity, and decreased renal glutathione reductase activity, Fig. 1; increased lipid peroxidation in liver and heart, Fig. 2) were not mimicked in galactosemic rats (although these values were higher in treated

TABLE I General characteristics of diabetic and galactose fed rats

| Sample | Body weight (g) | Liver weight (g) | Liver wt/ Body wt(%) | Glucose (mg/dL) | HbA1c % |
|---|---|---|---|---|---|
| Control (n = 15) | 342 ± 4 | 14 ± 0.3 | 4.0 ± 0.1 | 91 ± 5 | 1.8 ± 0.1 |
| STZ diabetic (n = 30) | *261 ± 7 | 13 ± 0.4 | *5.2 ± 0.1 | *592 ± 23 | *3.9 ± 0.2 |
| Insulin treated diabetic (n = 15) | 341 ± 4 | 13 ± 0.4 | 4.0 ± 0.1 | 160 ± 32 | 2.0 ± 0.1 |
| Galactose fed (n = 15) | *274 ± 5 | *11 ± 0.3 | 3.9 ± 0.1 | 80 ± 6 | 2.4 ± 0.1 |

Values are means ± s.e.m.
*$p < 0.05$ compared to controls.

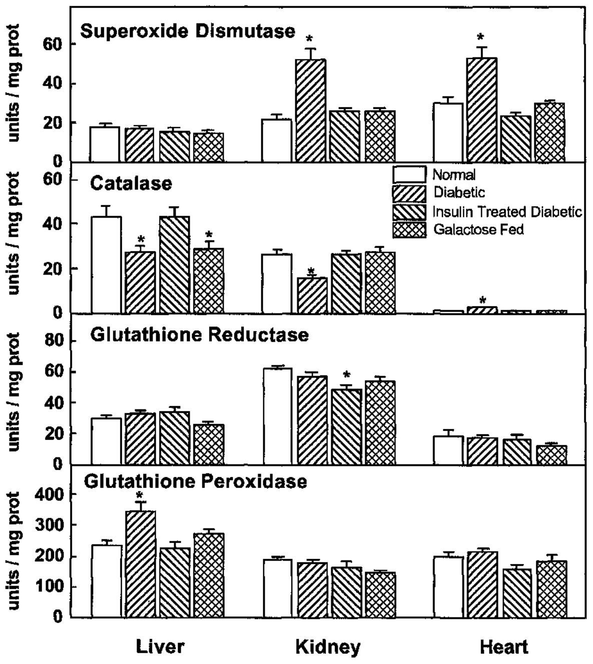

FIGURE 1 Activity of enzyme biomarkers of oxidative stress. Effects of 30 days of streptozotocin-induced diabetes, insulin-treated diabetes, and 50% galactose diet on enzyme activities in liver, kidney, and heart. One unit of superoxide dismutase is defined as the quantity of superoxide dismutase required to produce 50% inhibition of the rate of reduction of cytochrome C under assay conditions. One unit of catalase is defined as the amount of enzyme that liberates half the peroxide oxygen from a hydrogen peroxide solution in 100 seconds at 25°C. One unit of glutathione reductase and glutathione peroxidase is equivalent to one nmol NADPH oxidized per minute. Values represent means ± s.e.m. of 6 to 15 animals per group. *Significantly different from normal control, $p < 0.05$.

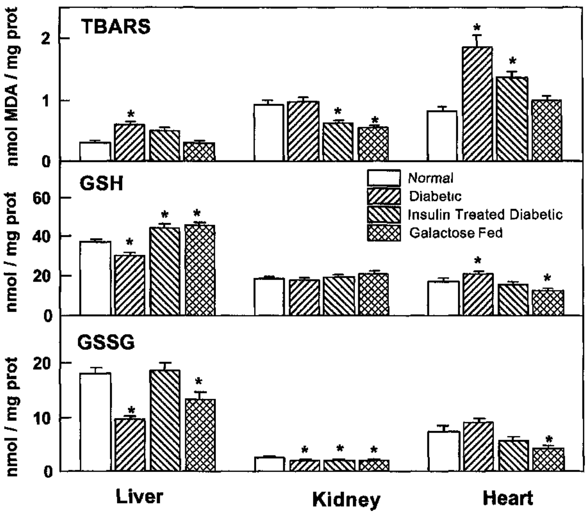

FIGURE 2 Concentrations of biomarkers of oxidative stress. Effects of 30 days of streptozotocin-induced diabetes, insulin-treated diabetes, and 50% galactose diet on concentrations of thiobarbituric acid reactive substances (TBARS), reduced glutathione (GSH) and oxidized glutathione (GSSG) in liver, kidney, and heart. Values represent means ± s.e.m. of 6 to 15 animals per group. *Significantly different from normal control, $p < 0.05$.

animals than in control rats). Curiously, GSH concentration in both liver and heart was affected in opposite ways in diabetic rats and galactose-fed rats (Fig. 2). Insulin treatment reversed/prevented the activity changes in renal and cardiac superoxide dismutase, renal and cardiac catalase, and hepatic glutathione peroxidase (Fig. 1). Interestingly, renal glutathione reductase after insulin administration was decreased below the levels in normal and untreated diabetic rats. As Figure 2 shows, insulin treatment of diabetic rats reversed/prevented the hepatic changes in TBARS, GSH and GSSG as well as the increase in cardiac GSH.

### DISCUSSION

Experimentally galactosemic animals have proven to be a productive model for the study of diabetic retinopathy, extensive studies having shown that the effects of high blood hexoses accurately mimic the morphological lesions of DM in the retina.[11,21] Galactose, flooding into the retinal tissue in excess, initiates non-enzymatic glycation and enters into the polyol pathway to generate free radicals and induce oxidative damage, as well as activating protein kinase C and other diabetes-like abnormalities.[7,22–25]

Further, therapeutics research has found this model to be useful in determining the utility of experimental drugs in controlling this exaggerated polyol pathway.[8] While antioxidants do not promise a cure for chronic complications of DM, many studies[26–32] have shown they have some success in ameliorating these complications *in vitro*. Some researchers have sought to utilize the experimentally galactosemic model for research into antioxidant therapies in the eye,[9,33] a viable strategy since previous research has so clearly shown the utility of this model in this tissue. However, galactosemic retinopathy does not always respond to treatment in the same way that diabetic rats do.[25]

The elegance of this model, its ability to isolate high blood hexoses and the sequelae of this hyperhexosemia from the other metabolic imbalances found in DM, makes it appealing to research outside of the realm of the eye. Some studies have begun to do just that, and have found that experimental galactosemia is indeed useful in antioxidant therapeutics research in other tissues, where the administration of a mixture of antioxidants did effectively control cardiac abnormalities in both diabetic and galactosemic animals.[31] However, in spite of these successes, this study urges caution in the expanded use of this model.

Galactose feeding does render the whole animal hypergalactosemic, not just the eye or the heart.[22] Yet, the responses of other tissues to excessive blood hexose have not been fully characterized. This study sought to evaluate oxidative stress as seen in this model, and did find some similarities between galactosemia and STZ-induced diabetes, particularly in the liver. However, interactions of glutathione in the two models are disparate. The concentration of hepatic GSSG and activity of glutathione peroxidase are both affected by diabetes but not by galactose feeding, and the effects of diabetes and galactose on the concentrations of GSH are opposed.

The overall level of concordance between the two groups is not convincing. Diabetes exerts an extensive systemic effect, acting at multiple sites with equal intensity. On the other hand, the galactose model relies on intestinal absorption of galactose, which then is passed *via* the portal circulation into the systemic circulation. The end result is that in the galactose-fed model, the liver is exposed to the highest concentration of the oxidative stressor and thus may bear the brunt of the damage. This could explain the significant similarities between the models in the liver, and the lack of consonance in the other organs.

The apparent lack of statistical significance in the elevated level of HbA1c in galactose-fed rats (Tab. I) is an artifact of the statistical analysis. If all four groups are analyzed, only the diabetic group is significantly different from normal ($p < 0.001$); the galactosemic group is not significantly different ($p > 0.05$). However, if only the normal and galactosemic groups are compared, then the galactosemic HbA1c rises to the level of significance ($p < 0.001$). In other studies where only these two groups are compared and where hyperglycemia is maintained for 12–22 months,[34,35] total glycated hemoglobin levels in the galactose-fed group appear to be elevated from normal levels.

Thus, in spite of some agreement, the composite picture presented in this study shows that galactose feeding does not render a model that closely mimics the effects of diabetes in extraocular tissues. It seems likely that hyperglycemia alone, as isolated in the galactosemia model, is not sufficient to explain the oxidative stress in liver, kidney and heart of diabetic rats. Further comparisons between the experimentally hypergalactosemic model and the diabetic model could focus on isolating other potential mechanisms of diabetic pathology, such as nonenzymatic protein glycation and exaggeration of the polyol pathway,[8,36,37] as well as examining specific cellular and molecular indices.

In conclusion, in light of the overarching divergence of biomarkers of oxidative stress between the models of the diabetic animal and the galactosemic animal, we agree with previous studies[38] that urge caution in the use of this model in studies of hyperglycemia as seen in DM.

### *References*

[1] Foster, D. W. (1998). Diabetes mellitus. In: *Harrison's Principles of Internal Medicine*, edited by Fauci, A. S., Braunwald, E., Isselbacher, K. J. and Wilson, J. D., pp. 2060–2081. McGraw- Hill, New York.

[2] Cerami, A., Vlassara, H. and Brownlee, M. (1988). Role of advanced glycosylation products in complications of diabetes, *Diabetes Care*, **11**(Suppl 1), 73–79.

[3] Brownlee, M., Cerami, A. and Vlassara, H. (1988). Advanced glycosylation end products in tissue and the biochemical basis of diabetic complications, *N. Engl. J. Med.*, **318**, 1315–1321.

[4] Hunt, J. V. and Wolff, S. P. (1991). Oxidative glycation and free radical production: a causal mechanism of diabetic complications, *Free Radic. Res. Commun.*, **12–13**, 115–123.

[5] Hunt, J. V., Dean, R. T. and Wolff, S. P. (1988). Hydroxyl radical production and autoxidative glycosylation. Glucose autoxidation as the cause of protein damage in the experimental glycation model of diabetes mellitus and ageing, *Biochem. J.*, **256**, 205–212.

[6] Baynes, J. W. (1991). Role of oxidative stress in development of complications in diabetes, *Diabetes*, **40**, 405–412.

[7] Berry, G. T. (1995). The role of polyols in the pathophysiology of hypergalactosemia, *Eur. J. Pediatr.*, **154**, S53–64.

[8] Lou, M. F., Dickerson, J. E. Jr., Chandler, M. L., Brazzell, R. K. and York, B. M. Jr. (1989). The prevention of biochemical changes in lens, retina, and nerve of galactosemic dogs by the aldose reductase inhibitor AL01576, *J. Ocul. Pharmacol.*, **5**, 233–240.

[9] Kowluru, R. A., Kern, T. S. and Engerman, R. L. (1997). Abnormalities of retinal metabolism in diabetes or experimental galactosemia. IV. Antioxidant defense system, *Free Radic. Biol. Med.*, **22**, 587–592.

[10] Engerman, R. L. and Kern, T. S. (1995). Retinopathy in animal models of diabetes, *Diabetes Metab. Rev.*, **11**, 109–120.

[11] Engerman, R. L. and Kern, T. S. (1984). Experimental galactosemia produces diabetic-like retinopathy, *Diabetes*, **33**, 97–100.

[12] National Research Council (1996). *Guide for the Care and Use of Laboratory Animals*. Washington DC, National Academy Press.

[13] Ohkawa, H., Ohishi, N. and Yagi, K. (1979). Assay for lipid peroxides in animal tissues by thiobarbituric acid reaction, *Anal. Biochem.*, **95**, 351–358.

[14] Luck, H. (1963). Catalase. In: *Methods of Enzymatic Analysis*, edited by Bergmeyer, H.-U., pp. 885–888. New York, Academic Press.

[15] Crapo, J. D., McCord, J. M. and Fridovich, I. (1978). Preparation and assay of superoxide dismutases, *Methods Enzymol.*, **53**, 382–393.

[16] Tappel, A. L. (1978). Glutathione peroxidase and hydroperoxides, *Methods Enzymol.*, **52**, 506–513.

[17] Carlberg, I. and Mannervik, B. (1975). Purification and characterization of the flavoenzyme glutathione reductase from rat liver, *J. Biol. Chem.*, **250**, 5475–5480.

[18] Hissin, P. J. and Hilf, R. (1976). A fluorometric method for determination of oxidized and reduced glutathione in tissues, *Anal. Biochem.*, **74**, 214–226.

[19] Lowry, O., Rosebrough, N. J., Farr, A. L. and Randall, R. J. (1951). Protein measurements with Folin phenol reagent, *J. Biol. Chem.*, **193**, 265–275.

[20] Isselbacher, K. J. (1998). Galactosemia, galactokinase deficiency, and other rare disorders of carbohydrate metabolism. In: *Harrison's Principles of Internal Medicine*, edited by Fauci, A. S., Braunwald, E., Isselbacher, K. J. and Wilson, J. D., pp. 2208–2209. New York, McGraw-Hill.

[21] Engerman, R. L. and Kern, T. S. (1986). Hyperglycemia as a cause of diabetic retinopathy, *Metabolism*, **35**, 20–23.

[22] Kern, T. S. and Engerman, R. L. (1996). Capillary lesions develop in retina rather than cerebral cortex in diabetes and experimental galactosemia, *Arch. Ophthalmol.*, **114**, 306–310.

[23] Glover, J. P., Jacot, J. L., Basso, M. D., Hohman, T. C. and Robison, W. G. (2000). Retinal capillary dilation: early diabetic-like retinopathy in the galactose-fed rat model, *J. Ocul. Pharmacol. Ther.*, **16**, 167–172.

[24] Robison, W. G., Jacot, J. L., Glover, J. P., Basso, M. D. and Hohman, T. C. (1998). Diabetic-like retinopathy: early and late intervention therapies in galactose-fed rats, *Invest. Ophthalmol. Vis. Sci.*, **39**, 1933–1941.

[25] Kowluru, R. A., Engerman, R. L. and Kern, T. S. (2000). Abnormalities of retinal metabolism in diabetes or experimental galactosemia VIII. Prevention by aminoguanidine, *Curr. Eye Res.*, **21**, 814–819.

[26] Cameron, N. E. and Cotter, M. A. (1993). Potential therapeutic approaches to the treatment or prevention of diabetic neuropathy: evidence from experimental studies, *Diabet. Med.*, **10**, 593–605.

[27] Gries, F. A. (1995). Alternative therapeutic principles in the prevention of microvascular and neuropathic complications, *Diabetes Res. Clin. Pract.*, **28**(Suppl.), S201–207.

[28] Giugliano, D., Ceriello, A. and Paolisso, G. (1996). Oxidative stress and diabetic vascular complications, *Diabetes Care*, **19**, 257–267.

[29] Hallberg, C. K., Trocme, S. D. and Ansari, N. H. (1996). Acceleration of corneal wound healing in diabetic rats by the antioxidant trolox, *Res. Commun. Mol. Pathol. Pharmacol.*, **93**, 3–12.

[30] Hammes, H. P., Bartmann, A., Engel, L. and Wulfroth, P. (1997). Antioxidant treatment of experimental diabetic retinopathy in rats with nicanartine, *Diabetologia*, **40**, 629–634.

[31] Kowluru, R. A., Engerman, R. L. and Kern, T. S. (2000). Diabetes-induced metabolic abnormalities in myocardium: effect of antioxidant therapy, *Free Radic. Res.*, **32**, 67–74.

[32] Maxwell, S. R. (1995). Prospects for the use of antioxidant therapies, *Drugs*, **49**, 345–361.

[33] Kern, T. S., Tang, J., Mizutani, M., Kowluru, R. A., Nagaraj, R. H., Romeo, G., Podesta, F. and Lorenzi, M. (2000). Response of capillary cell death to aminoguanidine predicts the development of retinopathy: comparison of diabetes and galactosemia, *Invest. Ophthalmol. Vis. Sci.*, **41**, 3972–3978.

[34] Kern, T. S. and Engerman, R. L. (1994). Comparison of retinal lesions in alloxan-diabetic rats and galactose-fed rats, *Curr. Eye Res.*, **13**, 863–867.

[35] Monnier, V. M., Sell, D. R., Abdul-Karim, F. W. and Emancipator, S. N. (1988). Collagen browning and cross-linking are increased in chronic experimental hyperglycemia. Relevance to diabetes and aging, *Diabetes*, **37**, 867–872.

[36] Robison, W. G., Tillis, T. N., Laver, N. and Kinoshita, J. H. (1990). Diabetes-related histopathologies of the rat retina prevented with an aldose reductase inhibitor, *Exp. Eye Res.*, **50**, 355–366.

[37] Tomlinson, D. R. (2000). Aldose reductase and tissue damage in diabetes. In: *Diabetic Retinopathy*, edited by Bijsterveld, O. P. v., pp. 189–200. London, Martin Dunitz.

[38] Willars, G. B., Lambourne, J. E. and Tomlinson, D. R. (1987). Does galactose feeding provide a valid model of consequences of exaggerated polyol-pathway flux in peripheral nerve in experimental diabetes? *Diabetes*, **36**, 1425–1431.

